# Implementation Effectiveness of a Parent-Directed YouTube Video (“It Doesn’t Have To Hurt”) on Evidence-Based Strategies to Manage Needle Pain: Descriptive Survey Study

**DOI:** 10.2196/13552

**Published:** 2020-03-04

**Authors:** Christine T Chambers, Justine Dol, Jennifer A Parker, Line Caes, Kathryn A Birnie, Anna Taddio, Marsha Campbell-Yeo, Scott A Halperin, Jennifer Langille

**Affiliations:** 1 Department of Psychology and Neuroscience Dalhousie University Halifax, NS Canada; 2 Department of Pediatrics Dalhousie University Halifax, NS Canada; 3 Centre for Pediatric Pain Research IWK Health Centre Halifax, NS Canada; 4 Faculty of Health Dalhousie University Halifax, NS Canada; 5 Division of Psychology Faculty of Natural Sciences University of Stirling Stirling United Kingdom; 6 Alberta Children's Hospital Calgary, AB Canada; 7 Department of Anesthesiology, Perioperative and Pain Medicine, Cumming School of Medicine University of Calgary Calgary, AB Canada; 8 The Hospital for Sick Children Toronto, ON Canada; 9 Leslie Dan Faculty of Pharmacy University of Toronto Toronto, ON Canada; 10 School of Nursing Dalhousie University Halifax, NS Canada; 11 Department of Pediatrics IWK Health Centre Halifax, NS Canada; 12 Canadian Center for Vaccinology IWK Health Centre Halifax, NS Canada

**Keywords:** pain management, child, knowledge translation, social media

## Abstract

**Background:**

Despite the availability of high-quality evidence and clinical practice guidelines for the effective management of pediatric pain, this evidence is rarely used in practice for managing children’s pain from needle procedures such as vaccinations. Parents are generally unaware of pain management strategies they can use with their children.

**Objective:**

This study aimed to develop, implement, and evaluate the implementation effectiveness of a parent-directed YouTube video on evidence-based strategies to manage needle pain in children.

**Methods:**

This was a descriptive study. Analytics were extracted from YouTube to describe video reach. A Web-based survey was used to seek parent and health care professional (HCP) feedback about the video. The 2-minute 18-second video was launched on YouTube on November 4, 2013. In the video, a 4-year-old girl tells parents what they should and should not do to help needles hurt less. The key evidence-based messages shared in the video were distraction, deep breathing, and topical anesthetic creams. A group of parents (n=163) and HCPs (n=278) completed the Web-based survey. Measures of reach included number of unique views, country where the video was viewed, sex of the viewer, and length of watch time. The Web-based survey assessed implementation outcomes of the video, such as acceptability, appropriateness, penetration, and adoption.

**Results:**

As of November 4, 2018 (5 years after launch), the video had 237,132 unique views from 182 countries, with most viewers watching an average of 55.1% (76/138 seconds) of the video. Overall, both parents and HCPs reported strong acceptance of the video (ie, they liked the video, found it helpful, and felt more confident) and reported significant improvements in plans to use distraction, deep breathing, and topical anesthetic creams.

**Conclusions:**

This parent-directed YouTube video was an acceptable and appropriate way to disseminate evidence about the procedure of pain management to a large number of parents.

## Introduction

### Background

Despite the availability of high-quality evidence and clinical practice guidelines for effective management of pediatric pain, best available evidence is rarely used in practice for managing children’s pain [1,2]. More than two-thirds of hospitalized children had no documented pain management intervention (pharmacological, psychological, or physical) for painful procedures [3]. Painful procedures are not limited to hospitalized children; even healthy children will receive up to two dozen needles before the age of 5 years [4]. Although simple, cost-effective, and evidence-based pain-relieving interventions exist for vaccination in school-aged children (eg, relaxation, distraction strategies, and topical anesthetic creams), fewer than 5% of children undergoing vaccination receive any form of pain management [4]. This is alarming as poorly managed pain is associated with a range of negative short- and long-term effects [5-8], including pain sensitization and development of needle fears and avoidance, which can contribute to vaccine hesitancy.

Existing studies of knowledge translation (KT; ie, a process that includes dissemination and application of scientific knowledge to improve health [9,10]) interventions in procedural pain management have primarily targeted health care professionals (HCPs) [11-14], yet parents can also serve as powerful and consistent pain management advocates for children [15]. A recent systematic review found that parents felt unsupported in taking an active role when their children are undergoing painful medical procedures [16]. In other health areas, interventions directed to patients have been more effective at improving outcomes than those directed to HCPs [17]. Knowledge synthesis research and clinical practice guidelines for procedural pain management in children include evidence-based recommendations that are simple for parents to implement [4,18-21]; however, most parents are unaware of these strategies [4].

Social media brings new opportunities for KT of health information to parents and an opportunity to reach a very large group of individuals. The use of social media is growing exponentially, with recent data showing that 65% of all adults now use social media (a 10-fold increase in the last decade) [22-27]. Parents are high users of social media (75% of parents use social media) [28] and are increasingly using social media for information about child health and parenting [24,28,29]. Yet, the quality of content shared about children’s health on the Internet is questionable at best [30-38]. Several YouTube videos have recently been developed and evaluated as dissemination tools to address procedural pain management in neonates with good success [39,40]. There is a need to develop high-quality, evidence-based, parent-targeted KT tools to share pediatric pain management techniques to improve outcomes for parents and children.

However, developing parent-targeted KT tools for pediatric pain management is not enough. It is important to evaluate the impact of the tool. Although traditional clinical trials focus on the efficacy of interventions in reducing pain in children, implementation studies focus on outcomes associated with the intervention that determine whether an intervention actually gets used. Implementation outcomes include acceptability (ie, the intervention is satisfactory), adoption (ie, intention or action to try or use an evidence-based practice), appropriateness (ie, perceived fit of the intervention to address a particular issue or problem), feasibility (ie, the extent to which the intervention can be carried out within a particular setting), fidelity (ie, the degree to which the intervention was implemented as prescribed by the original developers), implementation cost (ie, the financial impact of the intervention), penetration (ie, integration or spread of the intervention), and sustainability (ie, the extent to which the intervention is maintained or integrated into a service setting) [41].

### Objectives

The objective of this study was to develop, implement, and evaluate the implementation effectiveness of a parent-directed KT YouTube video to reach and inform parents on evidence-based strategies to manage needle pain in children. Video reach was measured using available Web-based analytics (ie, number of unique views, country where the video was viewed, sex of the viewer, and length of watch time) captured 5 years after the public launch of the video. The effectiveness of the video was assessed via several implementation outcomes (eg, acceptability, appropriateness, penetration, and adoption) among a sample of parents and HCPs who completed a Web-based survey.

## Methods

### “It Doesn’t Have To Hurt” Video Development

A brief (2 min 18 seconds) YouTube video for parents was developed to summarize evidence-based strategies for procedural pain management in a fun and entertaining way. The video [42] is hosted on the IWK Health Centre’s YouTube channel and was released on November 4, 2013. In the video, a 4-year-old girl tells parents what they should and should not do to help make needles hurt less. This video was the first in a video series to reach parents with evidence-based information about children’s pain (the second video was on neonatal pain management [39]).

Before the production of the video, a storyboard and a script were developed in collaboration with a communication company based on the synthesis of existing evidence-based information that was verified by the research team, partners, and parents. Initial drafts went through several rounds of revision and refinement before finalization. Once produced, the video was disseminated using a range of Web based and social media strategies, including emails, listservs, parenting forums, magazines, news coverage (television and newspaper), discussion groups, websites, blogs, social media, and other networking sites (eg, Twitter, Facebook, and LinkedIn). To further promote the video, posters, handouts, and social media images were also created and revised, and YouTube advertising campaigns and sponsored Facebook posts were used (see Multimedia Appendix 1 [43]). The target audience for the video was primarily parents with the goal of providing evidence-based strategies to manage needle pain in children. Secondary audiences included HCPs with whom parents would be interacting during painful procedures of their children, such as doctors and nurses.

Additional funding was obtained to repromote the video in February 2015. Initially produced in English, subtitles subsequently were created and uploaded for 16 languages, including Arabic, Chinese, Danish, Dutch, Finnish, French Canadian, German, Hungarian, Icelandic, Norwegian, Persian, Polish, Portuguese, Russian, Spanish, and Swedish. Through a partnership with the Quebec Pain Research Network, a French dubbed version of the video was released in April 2017 (Ça n’a pas besoin de faire mal—Conseils pour aider les enfants à recevoir une piqûre [44]), which has over 64,000 views to date. The goal of the translations was to enhance the reach of the video not only in Canada but also around the world. Only data from the English version of the video are reported here.

### Data Collection and Analysis

Reach data were collected through social media metrics available via YouTube analytics (ie, geography, view time, traffic source, watch devices, and close caption use). Although Google and its subsidiary YouTube do not publish the algorithms for generating their reports, YouTube analytics have been consistently used to report on reach statistics for videos [40]. Data were obtained from the IWK Health Centre’s YouTube channel reported from the initial launch to November 4, 2018 for a 5-year period. Data were tracked annually with the 5-year data extracted and summarized in this paper (because of the changes in YouTube analytic reporting, country data are based on 4-year data).

A Web-based survey was used to collect feedback from parents and HCPs. Viewers were prompted to complete an electronic survey after watching the video available through a link in the description text on YouTube as well as through the dissemination methods mentioned above. A total of two separate surveys were created: 1 for parents and 1 for HCPs. Participants who completed the surveys were self-selected from the viewers, with no inclusion or exclusion criteria stated with survey completion implying consent. Ethics approval for the survey was obtained from the Research Ethics Board of the IWK Health Centre; the survey was left open for 2 years (from November 4, 2013, to November 1, 2015).

The approximate survey completion time was less than 10 min. Questions were developed to discover acceptability, adoption, and feasibility of the evidence-based techniques shown in the video. The same survey was used to evaluate the newborn video [39]. Aside from dichotomous yes or no questions, the other closed-ended questions were asked on a 5-point Likert scale, where 1 was *not at all* and 5 was *very much*. The survey was assessed for content validity and is available in Multimedia Appendix 2 [45]. The parent and HCP surveys differed only with respect to 1 question regarding the ages of their children and the type of HCPs, respectively. Other general demographic questions common to both surveys were the respondents’ age, sex, and country. No personally identifying information were collected (eg, name and address), and no personal health information data were collected. Responses to open-ended questions were grouped using thematic analysis [46], and perceptions of use before and after watching the video were analyzed using the related samples McNemar test [47].

## Results

### Video Analytics

Five years after the launch, the video had a total of 237,132 unique views and had been viewed in 182 countries. The average view time was 1 min 16 seconds, which is 55.1% (76/138 seconds) of the video and corresponds to the end of the initial presentation of all evidence-based strategies by the little girl to her mother (ie, before recap). The United States (42.5%) and Canada (24.8%) had the most views, followed by Australia (10.2%), New Zealand (8.5%), and the United Kingdom (5.4%). Viewers were primarily females (53.3%), aged between 25 to 34 years (36.6%) and 35 to 44 years (37.5%). Traffic to the video was through YouTube advertising (122,788/237,132, 51.8%), searching on YouTube (28,155/237,132, 11.8%), an external link (28,004/237,132, 11.8%), a suggested video on YouTube (22,752/237,132, 9.6%), or other methods (35,433/237,132, 15.0%). The video was primarily watched on tablets (102,904/237,132, 43.4%), computers (67,564/237,132, 28.5%), mobile devices (62,726/237,132, 26.5%), or other devices (3,938/237,132, 1.6%). The video was shared directly from YouTube a total of 801 times through various channels, including copying the link (266/801, 33.2%), Facebook (122/801, 15.2%), WhatsApp (52/801, 6.5%), Twitter (48/801, 6.0%), and other (313/801, 39.1%). The video was watched without subtitles 91.5% (217,030/237,132) of the time, with Arabic (5,392/237,132, 2.3%), French (4322/237,132, 1.8%), Spanish (2739/237,132, 1.2%), Russian (2304/237,132, 1.0%), and Portuguese (1669/237,132, 0.7%) as the top 5 most selected closed caption subtitles.

**Figure 1:**
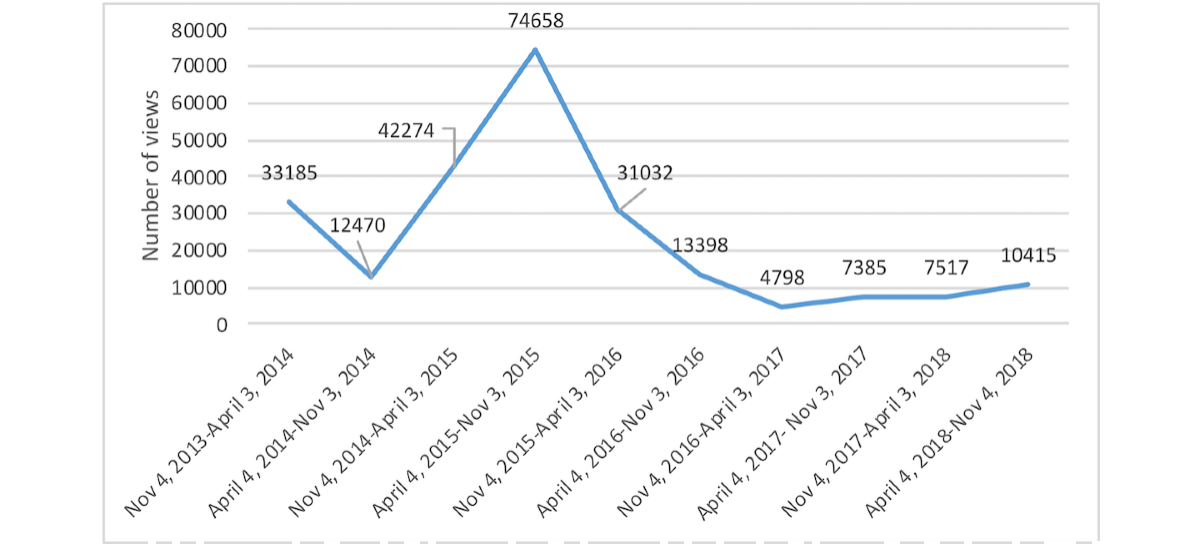
Number of views over a 5-year period.

Figure 1 shows the number of views the video received over time. The peak in views in 2015 was associated with additional formal promotion of the video at that time. Of note, no additional formal promotion of the video has been done since that time, so subsequent views (including 10,415 views during a 6-month period in 2018) are the result of organic sharing of the video.

### Survey Responses

Table 1 provides the demographics for the subsample of parents (n=163) and HCPs (n=278) who completed the survey. Overall, parents reported strong acceptance of the video (mean 4.06, SD 0.08), greater confidence in their ability to help their child cope (mean 3.62, SD 1.09), and that their child’s next needle procedure would be less painful (mean 3.31, SD 1.14; Table 2). Moreover, parents reported a significant increase in the likelihood of future use of pain management strategies such as deep breathing, topical anesthetic cream, and distraction (Table 3). Before watching the video, 84.6% (138/163) of parents reported that they had used reassuring phrases such as “it’ll be okay,” but after watching the video, 88.3% (144/163) of parents reported that they would avoid using those phases in the future. The majority of parents (110/163, 67.5%) reported that after watching the video, they were interested in learning more about how to manage children’s pain from needles, and 77.9% (127/163) of parents said that they planned to share the link with someone else, such as a family member or friend.

HCPs also reported strong acceptance of the video (mean 4.03, SD 1.09) and that the information obtained would be helpful to their practice (mean 3.53, SD 1.24; Table 2). Similar to parents, HCPs reported a significant increase in the likelihood of future use of deep breathing, topical anesthetic cream, and distraction (Table 3). However, the use of alternative interventions was reported by 31.7% (88/278) of HCPs, such as guided imagery, positions for comfort, and sucrose for infants. Before watching the video, 43.5% (121/278) of HCPs reported using reassuring phrases such as “it’ll be okay,” but after watching the video, 88.8% (247/278) of HCPs reported that they would avoid using those phrases in the future. After watching the video, HCPs reported that they felt more confident about helping children (mean 3.48, SD 1.24) and parents cope (mean 3.70, SD 1.18) with the pain and distress of getting a needle. The majority of HCPs (220/278, 79.4%) reported that after watching the video, they were interested in learning more about how to manage children’s pain from needles, and 74.1% (206/278) of HCPs said that they planned to share the link with someone else, such as a family member or friend.

**Table 1:** Survey demographics for parents and health care professionals.

Participants		Parents (n=163), n (%)	HCPs^a^ (n=278), n (%)
**Children^b^**			
	Baby (newborn to 12 months)	22 (9.2)	—^c^
	Toddler (1-2 years)	35 (14.6)	—
	Preschooler (3-4 years)	53 (22.2)	—
	School aged (5-12 years)	79 (33.1)	—
	Adolescent (13-17 years)	29 (12.1)	—
	Adult (18+ years)	21 (8.8)	—
**HCP occupation**			
	Nurse	—	117 (42.1)
	Physician (family, pediatrician, and other)	—	41 (14.7)
	Psychologist	—	52 (18.7)
	Child life specialist	—	30 (10.8)
	Other	—	38 (13.7)
**Location^d^**			
	Canada	118 (72.4)	128 (46.0)
	The United States	34 (20.9)	124 (44.6)
	Other	11 (6.7)	26 (9.4)
**Age^d^** **(years)**			
	Under 24	4 (2.5)	16 (5.8)
	25-35	39 (23.9)	66 (23.7)
	34-44	77 (47.2)	62 (22.3)
	44-54	32 (19.6)	67 (24.1)
	55-64	9 (5.5)	48 (17. 3)
	65 and above	—	12 (4.3)
**Sex^d^**			
	Woman	142 (87.1)	240 (86.3)
	Man	17 (10.4)	33 (11.9)

^a^HCP: health care professional.

^b^Parents could select more than 1 age group.

^c^Not applicable/no response.

^d^Percentage may not equal 100% as participants had the option to not answer.

**Table 2:** Acceptance of video by parents and health care professionals.

Acceptance of video		Response, mean (SD)^a^
**Parents (n=163)**		
	Liked the video	4.06 (0.80)
	Felt the video was helpful	3.90 (0.87)
	Felt more confident to help child cope	3.62 (1.09)
	Felt more confident that the next needle will be less painful	3.31 (1.14)
**Health care professionals (n=278)**		
	Liked the video	4.03 (1.09)
	Felt the video was helpful	3.53 (1.24)
	Felt more confident in their own practice	3.48 (1.24)
	Felt more confident in helping parents	3.70 (1.18)

^a^As reported on a 5-point scale, where 0=*not at all* and 5=*very much*.

**Table 3:** Past and predicted future use of techniques in the video by parents and health care professionals to reduce needle pain.

Predicted future use		Past, n (%)	Future, n (%)	Difference, %	*P* value
**Parents (n=163)**					
	Deep breathing or bubbles	25 (14.7)	89 (54.6)	39.9	<.001^a^
	Distraction	108 (66.3)	126 (77.6)	11.3	.04
	Topical anesthetic cream	31 (19.0)	102 (62.6)	43.6	<.001^a^
**Health care professionals (n=78)**					
	Deep breathing or bubbles	165 (59.4)	242 (87.1)	27.7	<.001^a^
	Distraction	230 (82.7)	245 (88.1)	5.4	<.05^a^
	Topical anesthetic cream	171 (61.5)	213 (76.6)	15.1	<.001^a^

^a^Significant differences.

### Qualitative Responses

When parents (n=42) and HCPs (n=98) were provided with the opportunity to offer an open-ended comment, seven themes arose: praise, suggestions for improvements, questions, plans to share video, identifying they already use these strategies, critiques about video or content, or other comments about thoughts and experiences. Multimedia Appendix 3 provides example comments from parents and HCPs in these categories as well as how they map onto implementation outcomes [41,48]. Of note, parents primarily praised the video, with comments including “great work—nice to see research move into practice” while also offering suggestions for the video, such as “It would have been helpful to actually see one of the techniques that you provided being used in the video.” Similarly, HCPs also shared praise of the video, such as “It is great to have a video as a tool to use with families…,” yet also had critiques of the video, such as “As a pediatric HCP for >25 years I object to the approach [referring to the exaggerate way in which the HCP indicated there would be needles today and put on gloves] that is displayed when the care provider comes into the room.”

## Discussion

### Principal Findings

This paper summarizes an evaluation of the implementation effectiveness of a parent-directed YouTube video about strategies parents can use to minimize their children’s procedural pain. The findings of this study suggest that the video is an acceptable method for disseminating evidence-based information regarding pain management with a high likelihood of adoption.

Penetration was high as the YouTube video received significant uptake during the period of analysis with 237,132 views. In similar parent-targeted pain management videos related to newborn pain, penetration rates measured at 12 months and 18 months had 65,478 views and 157,938 views, respectively, with the latter video part of this series receiving similar promotion as the current video [39,40]. Parents and HCPs both showed strong acceptance of the video and intention to adopt evidence-based pediatric pain management behaviors after watching the video. Parents reported significant increases in the degree to which they would use each of the pain management strategies demonstrated in the video, especially for deep breathing and topical anesthetic cream. Alternatively, HCPs reported smaller yet still significant increases in the degree to which they would use each of the pain management strategies. Generally, parents and HCPs were receptive to the evidence-based information provided through the video and were willing to use it to minimize pain, which suggests that the video was effective in gaining acceptability and adoption among parents and HCPs.

Most parents and HCPs felt that the strategies were appropriate to minimize child pain during immunization. However, some concerns related to the feasibility of the strategies were noted in comments from parents and HCPs. One-third of the parents commented that the strategies provided were not practical (ie, topical creams may not be available and unsure as to how long a child should use it), whereas nearly half of all HCPs identified similar barriers, such as cost and application timing for the topical anesthetic cream. Parents also commented on concerns related to their HCP’s acceptance of the strategies: “I’m not 100% sure my doctor would be ok with us blowing bubbles at this office.” This was reflected in some HCP responses as well: “may find other way to encourage deep breathing to avoid ‘bubble mess’.”

In terms of the costs associated with this video, it cost Can $10,000 to professionally develop and produce this video and an additional Can $5000 to promote the video. It took considerable effort on the part of the team to promote the video, and further efforts in this area could be improved via partnering with parenting media who already have expertise in creating parenting content and an established reach to parents. Given the cost of video development and promotion in finances and time, this may be a hindrance for individual researchers or health care providers to undertake alone. However, consideration should be undertaken by public health organizations or clinical practice guideline developers that may have the financial resources and desire to create parent-targeted content. On the basis of this study and previous work, parent-targeted videos have the ability to reach a large number of people [39,40] and show evidence of impacting behavior change [49].

### Limitations

This study had a number of limitations. First, those parents and HCPs who completed our Web-based survey may not be representative of all parents and HCPs; they may represent groups of individuals who had a particular interest in or willingness to adopt pain management strategies. Second, our study did not examine the actual implementation of pain management strategies by parents and HCPs; we simply assessed their report of whether they would use the strategies. Future prospective research should follow parents and HCPs over time to determine whether the strategies were effectively implemented. Finally, our reach analytics were limited in our reliance on YouTube analytic reports for viewer data (which require viewers to be logged into their personal account to pull demographics on sex and age).

### Comparison With Prior Work

Communicating evidence-based recommendations for pain management to parents could be powerful, yet large-scale efforts to disseminate this knowledge to parents are lacking. A recent randomized trial showed that parental exposure to a 5-min educational video significantly increased parental pain management behaviors and decreased child pain [49]. However, the video in this trial was developed for research purposes and is not publicly available to parents.

Building on previous work evaluating publicly available YouTube videos directed at parents for pain management [39,50], this work is the first 5-year evaluation. Previous work has limited evaluation of the YouTube video to 12 months [40] and 18 months [39], leading to the current video having more views because of being available longer. In both previous studies, surveys were conducted with parents and HCPs and also found an increased likelihood of parents and HCPs using the techniques provided in the videos during the next painful procedure [39,40]. What is unique about the current video is its focus on children, whereas the other 2 videos targeted the newborn population, filling a gap in the evidence-based information available to parents on YouTube regarding pain management for procedures.

As identified in an earlier scoping review on publicly available videos on pediatric needle pain management [51], there is a continued need to evaluate the effectiveness of these videos. An effective implementation needs to include and illustrate acceptability, adoption, appropriateness, feasibility, fidelity, implementation cost, penetration, and sustainability [41]. Evaluation of the current video illustrates that the video was perceived as acceptable as illustrated by the positive response from parents and HCPs. It also illustrated the likelihood of adoption of behaviors as they were appropriate and feasible to implement by parents. This analysis also illustrates that it was able to achieve penetration with the significant number of views, and this was sustained over time. However, challenges remain in evaluating the true behavior change impact of this KT video as parents were not followed up to determine if parents or HCPs actually used the pain management strategies at a future appointment.

### Conclusions

This YouTube video benefited knowledge users, in this case, parents and HCPs, directly by providing them with evidence-based information about pain management for procedures in an engaging and accessible way. Owing to the dissemination method (ie, making the video openly available free of charge on YouTube), the video reached a very wide, international audience. Making evidence-based information on pediatrics available in this way has the potential to result in improved awareness and use of evidence-based practices, including, in this case, reduced pain and distress in children undergoing painful medical procedures and their families. Given the evidence of success in parent-targeted videos to improve pain management, opportunities exist to expand this work to other areas of pediatric health. Future research should consider conducting randomized controlled trials that explore the impact of such videos on behavior change outcomes.

## Appendix

Multimedia Appendix 1 Advertisement for promotion of video.

Multimedia Appendix 2 Surveys.

Multimedia Appendix 3 Qualitative findings from parents’ and health care professionals’ comments.

## Abbreviations

HCP: health care professional
KT: knowledge translation
