# Supplementary material for: Implementation Effectiveness of a Parent-Directed YouTube Video (“It Doesn’t Have To Hurt”) on Evidence-Based Strategies to Manage Needle Pain: Descriptive Survey Study
Source: JMIR Pediatr Parent. 2020 Mar 4;3(1):e13552. doi: 10.2196/13552 (PMC7081136; doi:10.2196/13552)

Multimedia Appendix 1: Advertisement for promotion of video

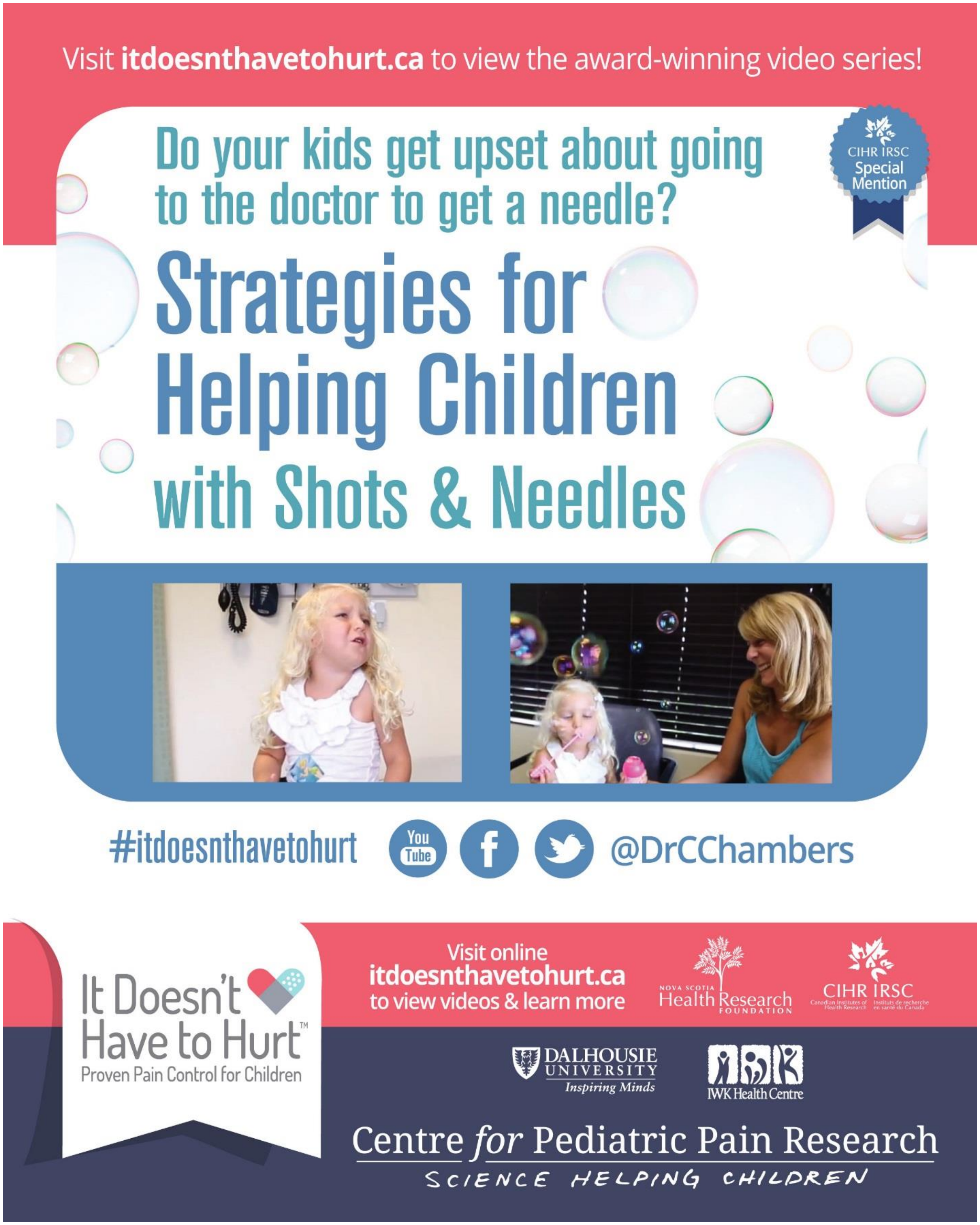

Supplement: Multimedia Appendix 1 [file pediatrics_v3i1e13552_app1.pdf]
