# Supplementary material for: Implementation Effectiveness of a Parent-Directed YouTube Video (“It Doesn’t Have To Hurt”) on Evidence-Based Strategies to Manage Needle Pain: Descriptive Survey Study
Source: JMIR Pediatr Parent. 2020 Mar 4;3(1):e13552. doi: 10.2196/13552 (PMC7081136; doi:10.2196/13552)
Supplement: Multimedia Appendix 2 [file pediatrics_v3i1e13552_app2.pdf]

Multimedia Appendix 2: Surveys

Parent Survey

1. How did you find this video?
   a. Searching YouTube
   b. Family/Friend
   c. Health Care Professional
   d. Email
   e. Facebook
   f. Twitter
   g. IWK Website
   h. Pediatric-Pain.ca (CPPR) website
   i. Other website (describe below)
   j. Other (describe below)

2. How old are your children (check all that apply)?
   a. Baby (newborn – 12 months old)
   b. Toddler (1-2 years old)
   c. Preschooler (3-4 years old)
   d. School-aged (5-12 years old)
   e. Adolescent (13-17 years old)
   f. Adult (18+ years old)

3. Overall, how much did you like this video?
   a. 1 – not at all
   b. 2
   c. 3
   d. 4
   e. 5 – Very much

4. Overall, how helpful was this video to you?
   a. 1 – not at all
   b. 2
   c. 3
   d. 4
   e. 5 – Very much

5. Before watching this video, have you/your family ever used any of these techniques to reduce your child's needle pain?
    a. Blowing bubbles/deep breathing
    b. Distraction with books, videos, songs, toys, etc
    c. ELMA or other topical anesthetic cream
    d. I use (an)other technique(s) (describe below)

6. Before watching this video, when your child had a needle procedure have you said reassuring phrases like, "don't worry" and/or "It'll be okay"?
    a. Yes
    b. No

7. After watching this video, do you/your family plan to use (or ask your doctor/nurse if you could use) any of these techniques during your child's next needle?
    a. Blowing bubbles/deep breathing
    b. Distraction with books, videos, songs, toys, etc
    c. ELMA or other topical anesthetic cream
    d. I would not use any of these techniques (if you could not use any of these techniques, please let us know why?)

8. After watching this video, will you try to avoid using reassuring phrases, like "don't worry and "it will be okay" during your child's next needle procedure?
    a. Yes
    b. No

9. After watching this video, do you feel more confident about how you can help your child cope with the pain and distress of getting a needle?
    a. 1 – not at all
    b. 2
    c. 3
    d. 4
    e. 5 – Very much

10. After watching this video, do you feel more confident that your child's next needle experience will be less painful and distressing?
    a. 1 – not at all
    b. 2
    c. 3
    d. 4
    e. 5 – Very much

11. After watching this video, are you interested in learning more about ways to decrease your child’s needle pain (eg, through websites and other videos)?
    a. Yes
    b. No

12. Did/Would you send the link to this video to anyone (family, friends, healthcare providers)?
    a. Yes
    b. No

13. What country are you from?

14. How old are you?

15. What sex are you?

16. Is there anything else you would like us to know?

Healthcare providers Survey

1. How did you find this video?
    a. Searching YouTube
    b. Family/Friend
    c. Health Care Professional
    d. Email
    e. Facebook
    f. Twitter
    g. IWK Website
    h. Pediatric-Pain.ca (CPPR) website
    i. Other website (describe below)
    j. Other (describe below)

2. What type of clinician/health care provider are you?
    a. Nurse
    b. Family physician
    c. Pediatrician
    d. Other physician
    e. Psychologist
    f. Psychiatrist
    g. Pharmacist
    h. Dentist
    i. Other:

3. Overall, how much did you like this video?
    a. 1 – not at all
    b. 2
    c. 3
    d. 4
    e. 5 – Very much

4. Overall, how helpful was this video to you and your practice?
    a. 1 – not at all
    b. 2
    c. 3
    d. 4
    e. 5 – Very much

5. Before watching this video, in your practice, do you use (or encourage parents to use) any of the following strategies to reduce children's needle pain? (check all that apply)?
    e. Blowing bubbles/deep breathing
    f. Distraction with books, videos, songs, toys, etc
    g. ELMA or other topical anesthetic cream
    h. I use (an)other technique(s) (describe below)

6. Before watching this video, in your practice, have you said reassuring phrases to children receiving needles like, "don't worry" and/or "It'll be okay"?
    a. Yes
    b. No

7. After watching this video, which of these techniques will you encourage children and parents to use to reduce needle pain (check all that apply)?
    a. Blowing bubbles/deep breathing
    b. Distraction with books, videos, songs, toys, etc
    c. ELMA or other topical anesthetic cream
    d. I would not use any of these techniques (if you could not use any of these techniques, please let us know why?)

8. After watching this video, will you try to avoid using reassuring phrases, like "don't worry and "it will be okay" during your next needle procedure with a child?
    a. Yes
    b. No

9. After watching this video, do you feel more confident about helping children cope with the pain and distress of getting a needle?
    a. 1 – not at all
    b. 2
    c. 3
    d. 4
    e. 5 – Very much

10. After watching this video, do you feel more confident about how you can help parents help their children cope with the pain and distress of getting a needle?
    a. 1 – not at all
    b. 2
    c. 3
    d. 4
    e. 5 – Very much

11. After watching this video, are you interested in learning more about ways to decrease children’s needle pain (eg, through websites and other videos)?
    a. Yes
    b. No

12. Did/Would you send the link to this video to anyone (family, friends, healthcare providers)?
    a. Yes
    b. No

13. What country are you from?

14. How old are you?

15. What sex are you?

16. Is there anything else you would like us to know?
