# Supplementary material for: Implementation Effectiveness of a Parent-Directed YouTube Video (“It Doesn’t Have To Hurt”) on Evidence-Based Strategies to Manage Needle Pain: Descriptive Survey Study
Source: JMIR Pediatr Parent. 2020 Mar 4;3(1):e13552. doi: 10.2196/13552 (PMC7081136; doi:10.2196/13552)
Supplement: Multimedia Appendix 3 [file pediatrics_v3i1e13552_app3.pdf]

Multimedia Appendix 3. Qualitative findings from parents' and health care professionals' comments.

Theme	Implementation Outcome(s)	Quote - Parents	Quote - Health Care Professionals
Praise	Acceptability and adoption	• "Great work—nice to see research move into practice!"<br>• "This video has made a huge difference for our family. Thank you so much!"	• "The video is excellent...concise and to the point..."<br>• "It is great to have a video as a tool to use with families..."
Suggestions for the video	Appropriateness	• "It would have been helpful to actually see one of the techniques that you provided being used in the video."<br>• "Perhaps videos with children of different ages would be helpful and specific things parents and health practitioners can say—not just what	• "My suggestions perhaps detract from this, but here they are: 1) Maybe specify the age range to which this video applies and 2) endorse other strategies for other age ranges, e.g., breast feeding

Theme	Implementation Outcome(s)	Quote - Parents	Quote - Health Care Professionals
		they shouldn't say—would be helpful."	for infants, something other than bubble blowing for older children."<br>• "It would be nice to have more of an explanation for why reassurance isn't effective."
Critiques about video or content	Appropriateness and feasibility	• "There's often not time to do distracting games or bubbles beforehand."<br>• "It seems strange to me to just simply not talk about it. What should you say?"	• "I would have liked to see the techniques shown in more detail on the video not just saying that you can use them."<br>• "I disagree with the recommendation

Theme	Implementation Outcome(s)	Quote - Parents	Quote - Health Care Professionals
			for EMLA or similar cream on the video."<br>• "The little girl was a little hard to understand at times, so I am glad that the dialogue was also written."<br>• "EMLA patch is cost prohibitive for most parents, and placement on the correct site could be a problem."
Questions	Feasibility	• "Where the topic cream is available and whether the doctor provides it or the family."	• "I am not clear as to why you can't use the terminology 'it

Theme	Implementation Outcome(s)	Quote - Parents	Quote - Health Care Professionals
		• "What can we do as parents when the medical professionals use the 'it's gonna be ok' and don't provide us and our kids with any real comfort?!"	will be okay'...why not?"<br>• "Hard not to use phrases of reassurance. What is the rationale for NOT using them, if you are also doing the 'to-do's'?"
Plans to share video	Penetration	• "I think I will be using these techniques with my grandchildren and sharing the video with my children and friends."<br>• "I'll share with friends for sure!"	• "I have passed it on to some parents who might distribute it among their network."<br>• "All my parents should watch this video before their kids immunization visits!"

Theme	Implementation Outcome(s)	Quote - Parents	Quote - Health Care Professionals
Identifying they already use these strategies	Penetration and sustainability	• "I was aware of distraction techniques prior to this video..."<br>• "I've had IV needles inserted both with and without and know damned well Emla works pretty well."	• "I am a pediatric anesthesiologist and pain physician and have tried to encourage pediatricians to use these types of techniques for years."<br>• "Because of my profession and training, I already did these things."
Other comments about thoughts and experiences	Feasibility, acceptability, fidelity, and penetration	• "The majority of parents I know WOULD have naturally used phrases like 'it will be over soon' etc., just by instinct."<br>• "I attempted to purchase EMLA cream from my	• "I have been practicing both child life and education for over 35 years and I wish very much that information

Theme	Implementation Outcome(s)	Quote - Parents	Quote - Health Care Professionals
		pharmacy (Shoppers Drug Mart) before my son's 5 year old vaccinations and was told that it was not recommended for this use because it would enter the body through the puncture rather than staying topical. They would not sell it to me."<br>• "I'm not 100% sure my doctor would be ok with us blowing bubbles at this office."	was made more accessible to parents so their children would have pain free procedures."<br>• "I am not sure why there is so much resistance to topical anesthetics and distraction."<br>• "[I] may find other way to encourage deep breathing to avoid 'bubble mess'."
